# Using whole genome sequencing to study American foulbrood epidemiology in honeybees

**DOI:** 10.1371/journal.pone.0187924

**Published:** 2017-11-15

**Authors:** Joakim Ågren, Marc Oliver Schäfer, Eva Forsgren

**Affiliations:** 1 Department of Microbiology, National Veterinary Institute, Uppsala, Sweden; 2 Federal Research Institute for Animal Health, Friedrich-Loeffler-Institut, Greifswald, Insel Riems, Germany; 3 Department of Ecology, Swedish University of Agricultural Sciences, Uppsala, Sweden; Philipps-Universitat Marburg Fachbereich Biologie, GERMANY

## Abstract

American foulbrood (AFB), caused by *Paenibacillus larvae*, is a devastating disease in honeybees. In most countries, the disease is controlled through compulsory burning of symptomatic colonies causing major economic losses in apiculture. The pathogen is endemic to honeybees world-wide and is readily transmitted via the movement of hive equipment or bees. Molecular epidemiology of AFB currently largely relies on placing isolates in one of four ERIC-genotypes. However, a more powerful alternative is multi-locus sequence typing (MLST) using whole-genome sequencing (WGS), which allows for high-resolution studies of disease outbreaks. To evaluate WGS as a tool for AFB-epidemiology, we applied core genome MLST (cgMLST) on isolates from a recent outbreak of AFB in Sweden. The high resolution of the cgMLST allowed different bacterial clones involved in the disease outbreak to be identified and to trace the source of infection. The source was found to be a beekeeper who had sold bees to two other beekeepers, proving the epidemiological link between them. No such conclusion could have been made using conventional MLST or ERIC-typing. This is the first time that WGS has been used to study the epidemiology of AFB. The results show that the technique is very powerful for high-resolution tracing of AFB-outbreaks.

## Introduction

*Paenibacillus larvae* is a Gram-positive, spore-forming bacterium that causes American foulbrood disease (AFB) in honeybee larvae [1,2]. AFB is lethal and highly infectious to individual larvae. It can also be fatal to colonies and can easily be spread between apiaries unless proper control measures are carried out. AFB is highly contagious and the spread depends largely on apiculture; transportation and trading of bee material, hive equipment and honey both locally and globally [3]. In Sweden and many other countries, AFB is a notifiable disease and control measures like burning infected colonies and hive material or shaking the bees onto new wax foundations is required. The disease is widely distributed across the world causing great economic losses in apiculture [4] and is also widespread in most parts of southern and central Sweden; in northern Sweden it occurs only locally. The incidence of AFB in Sweden has decreased drastically during the second half of the 1900s, coinciding with the introduction of a strict control program aimed at preventing epidemic spread. However, it still causes local problems in parts of the country.[5]

Little is known about the genetic variation and population structure of *P*. *larvae*, information that could shed light on the observed patterns of disease. Molecular characterization, or sub-typing techniques with various discriminatory powers have been used to differentiate between strains and genotypes of *P*. *larvae* and helped unravel the epidemiology of AFB [6–8]. One major sub-typing technique is based on repetitive elements within bacterial genomes (rep-PCR) using enterobacterial repetitive intergenic consensus (ERIC) primers [7]. Four distinct genotypes (ERIC I-IV) have been identified *for P*. *larvae* using rep-PCR. These have further been shown to form two clusters when using pulsed-field gel electrophoresis (PFGE) [1]. PFGE has a higher resolution than regular gel electrophoresis and therefore adds discriminatory power to epidemiological studies of AFB [6]. Another typing scheme developed recently for *P*. *larvae* is multilocus sequence typing (MLST), which was used to determine the distribution and biogeography of *P*. *larvae* strains from 6 continents [8]. Although the main aim of the study was not epidemiological, a sub-set of data from an AFB outbreak in Jersey (an island in the English Channel) included in the study demonstrated the potential power of MLST in studies of disease transmission [8].

MLST has been somewhat of a typing gold standard for several pathogens, *e*.*g*. the *Bacillus cereus*-group [9] and *Campylobacter* spp. [10]. However, a traditional MLST based on 7 genes does not have enough resolution to enable source tracing of bacterial pathogens through an epidemic. Seven MLST-genes do not mutate fast enough to generate the variation required to distinguish alternative transmission scenarios [11,12]. Traditional MLST is a technique best suited for studying population structure of bacteria.

Since their emergence about 15 years ago, Next Generation Sequencing (NGS) technologies have radically changed infectious disease research. NGS has a broad range of applications including research, clinical diagnostics and epidemiology. Outbreak investigations and surveillance of communicable diseases have greatly improved by the higher resolution of NGS compared to other typing methods [13–15]. High-resolution epidemiological studies using Whole Genome Sequencing (WGS) can be conducted using either a single nucleotide polymorphism (SNP) approach or a gene-by-gene approach. The latter is essentially an MLST-type analysis, but extended to the whole genome [11,16]. New genomes are queried using an online reference database; any new alleles will get unique identifiers and the database will cover more and more of the allelic diversity of the organism. This way the allele nomenclature is standardized and new genomes can be compared to all previously analyzed and deposited genomes [11]. At the time writing, the publicly accessible online reference database for *P*. *larvae* MLST typing (PubMLST, http://pubmlst.org/plarvae/) covered only the seven standard MLST-genes.

WGS has been successfully used for epidemiological investigations of highly clonal and invariant bacterial species, *e*.*g*. *Bacillus anthracis* [14] and *Mycobacterium tuberculosis* [17], due to the ability of WGS to identify even minor variation. WGS technology is also valuable in retrospective studies for elucidating the start, progress and end of a disease outbreak, information which is useful for mitigating future outbreaks. WGS can also be used in real-time, to help monitor and control an ongoing outbreak [18].

Here we report the first use of WGS on *P*. *larvae* for an epidemiological application. Since AFB is mainly spread through beekeeping activities such as moving contaminated hive material, WGS could be a useful tool to determine when and from where the pathogen was introduced in a geographical area, a beekeeping operation or an apiary.

The aim of the study was to *i*) evaluate the use of core genome MLST to differentiate isolates of *P*. *larvae* and *ii*) to investigate in detail the epidemiology of an isolated AFB outbreak on the Baltic Isle of Gotland, Sweden.

## Materials and methods

No specific permissions were required for these locations/activities. The honeybee samples were collected by bee inspectors authorized by the local County Boards and the Swedish Board of Agriculture and sent to the National Reference Laboratory for Bee Health at the Swedish University of Agricultural Sciences. The field studies did not involve endangered or protected species, only managed honeybees (*Apis mellifera*).

### Sample origins

Samples of >100 adult worker bees were collected by bee inspectors from a total of 115 suspected *P*. *larvae* contaminated colonies in 42 apiaries on the Baltic island Gotland. An additional 28 *P*. *larvae*-positive samples from 16 apiaries across two large beekeeping operations in Uppland county on mainland Sweden obtained from previous unconnected AFB outbreaks in 2012 and 2014 respectively, were used to construct a local reference database for natural *P*. *larvae* whole-genome diversity in Sweden.

### Sample processing and microbial assays

Crude extracts from the bee samples were cultivated on MYPGP-agar plates as described previously [19]. In brief, 100 worker bees were crushed in a filter grinding bag (Neoreba®) with 20 ml of sterile 0.9% NaCl. The filtered extract was centrifuged for 10 minutes at 27,000 g. The resulting pellet was re-suspended in 2 ml sterile 0.9% NaCl, incubated at 85°C for 10 minutes and 10 μl extract was spread directly onto MYPGP-agar plates; 3 plates per isolate. The plates were incubated at 35°C in 5% CO_2_ for 7 days and the numbers of *P*. *larvae* colonies were counted. The data was presented as colony forming units (CFU) per bee. Material from symptomatic larvae in brood samples were streaked onto MYPGP-agar plates, incubated at 35°C in 5% CO_2_ for 7 days and *P*. *larvae* isolates stored at -20°C until further analyzed.

### ERIC-typing

All *P*. *larvae*-positive isolates were grown on MYPGP-agar and incubated at 35°C and 5% CO_2_ for 7 days [20]. DNA was extracted from the bacteria using the DNeasy® Blood & Tissue Kit (Qiagen, Hilden, Germany) following the manufacturer’s protocol for Gram-positive bacteria, eluting the DNA in 100 μl water. The DNA concentration and purity was determined using NanoDrop. The DNA samples were diluted with ultrapure water to a constant 20 ng/μl prior to their use in the molecular assays. Genotyping of the isolates by rep-PCR with ERIC primers [21] was performed as previously described [1]. The primer sequences used for DNA fingerprinting were ERIC1R: 5’-ATG TAA GCT CCT GGG GAT TCA C-3’ and ERIC2: 5’-AAG TAA GTG ACT GGG GTG AGC G-3’ [21]. The PCR reactions were performed in 25 μl total volume containing 1x HotStarTaq Master Mix (Qiagen), 2.25 mM MgCl_2_, 0.3 μM of each primer and 5 μl DNA template (20 ng/ μl). The PCR reaction conditions were as follows: 95°C, 15 min (sterilization and Taq activation), followed by 35 cycles at 94°C for 60 s, 53°C for 60 s and 72°C for 2.5 min, followed by a final elongation step at 72°C for 10 min. About 20 μl of each PCR reaction was analyzed on a 0.8% agarose gel. The DNA bands were stained with ethidium bromide (0.5 μg/ml) and visualized by UV light (302 nm). A collection of commercially available *P*. *larvae* reference strains were used as standards (DSM 25719, DSM7030 and CCUG48978 for ERIC I; DSM 25430, DSM16116 and CCUG48972 for ERIC II; LMG16252 for ERIC III; DSM3615 for ERIC IV).

### Genome sequencing

The exact DNA-concentrations of the samples were determined with the Qubit HS DNA-kit (Life technologies, Carlsbad, CA, USA). The sequencing libraries were prepared using the Nextera XT kit (Illumina, San Diego, CA, USA) and 250 bp paired-end sequencing was performed on a MiSeq sequencer (Illumina). The reads (down-sampled to 90x coverage) were assembled using the SPAdes v. 3.9.1 assembler with the ‘—careful’ option [22]. The assemblies were corrected using the Pilon 1.21 software with default settings [23]. The sequence reads from all isolates included in the study have been submitted to the European Nucleotide Archive (ENA) under the study accession number PRJEB12365.

### Standard 7-gene MLST

Traditional 7-gene MLST-analysis was performed by uploading the genomes to the PubMLST-database (http://pubmlst.org/plarvae) which returned the ST based on the standard MLST-scheme [8].

### Whole genome and core genome MLST

The phylogenetic relationships between the genomes were analyzed using the whole genome MLST (wgMLST) and core genome MLST (cgMLST) analysis software SeqSphere+ v3.1.0 (Ridom GmbH, Münster, Germany). Two target gene sets for cgMLST were determined by the MLST+ target definer function of SeqSphere+ with default parameters, using the genomes of *P*. *larvae* ATCC 9545 (ERIC I: NCBI accession NZ_CP019687.1) and DSM 25430 (ERIC II: NCBI accession NC_023134) as references for ERIC I and ERIC II genomes, respectively. Genes not suitable for cgMLST (*e*.*g*. multiple copy-genes) were filtered out using the software’s default settings. This exercise yielded 3,367 genes for ERIC I (out of a total of 4,460 genes in the ATCC 9545 genome) and 3,373 genes (out of a total of 4,060 genes in the DSM 25430 genome) for querying the sequenced outbreak isolates (S1 and S2 Tables). Minimum spanning trees were created based on the allelic differences between the outbreak genomes. The trees were enhanced in Adobe Illustrator (Adobe Systems Software) for presentation.

## Results

In the summer of 2014, symptoms of AFB were observed in a honeybee colony in a previously disease-free area on the island Gotland in the Baltic Sea. The colony was burned in accordance with Swedish legislation and the remaining colonies in the infected apiary and in other apiaries within a 3 km radius were inspected. No other diseased colonies were found upon visual inspection. However, since the owner of the infected apiary (Beekeeper 1) had other apiaries scattered throughout the island and had sold bee material (nukes) to other beekeepers, an additional 42 apiaries that were either owned by or contained bees sold by the affected beekeeper were investigated for the presence of *P*. *larvae*, using culture-based microbiological assays on extracts of adult bee samples [19,24].

Clinical symptoms of AFB were observed by visual inspection in 7 colonies in 4 of the 42 investigated apiaries on Gotland. In the bee samples collected in connection with the Gotland outbreak (G), high levels (>6,000 spores per bee) of *P*. *larvae* were detected in 24 colonies in 10 apiaries. *Paenibacillus larvae* was not detected by culture-based assays in any of the other sampled apiaries (n = 38) on the island. A total of 24 *P*. *larvae* isolates from the Gotland outbreak were recovered from diseased honeybee brood or adult bees (beekeepers 1–3, Table 1). The additional 28 *P*. *larvae* isolates from the county Uppland on the mainland (U) were isolated from adult bee samples from 28 colonies in 16 apiaries in 2 beekeeping operations the season after observed clinical disease (in 2014 and 2012 respectively, Table 1).

**Table 1:** Isolates included in the study, year of sampling, beekeeper, geographic and biological origin, ERIC-typing and 7-gene MSLT results. Isolates from Gotland are marked with the prefix “G” and isolates from Uppland with the prefix “U”.

					*Paenibacillus larvae* isolated from:			
Isolate	Year	Beekeeper	Apiary	Region	Adult bees	Symptomatic brood	ERIC-type	7-gene MLST type
G1	2014	1	1–1	Gotland		+	I	15
G2	2014	1	1–2	Gotland	+		I	15
G3	2014	1	1–2	Gotland	+		I	19
G4	2014	1	1–3	Gotland	+		I	15
G5	2014	1	1–3	Gotland	+		I	19
G6	2014	1	1–3	Gotland	+		I	15
G7	2014	1	1–3	Gotland	+		I	15
G8	2014	1	1–3	Gotland	+		I	19
G13	2014	1	1–4	Gotland	+		II	11
G14	2014	1	1–5	Gotland	+		I	15
G18	2014	1	1–6	Gotland	+		II	11
G19	2014	1	1–7	Gotland	+		I	19
G9	2014	1	1–8	Gotland	+		I	19
G23	2014	1	1–8	Gotland		+	II	11
G24	2014	1	1–8	Gotland		+	I	19
G10	2014	2	2–1	Gotland	+		II	11
G15	2014	2	2–1	Gotland	+		II	11
G16	2014	2	2–1	Gotland	+		II	11
G17	2014	2	2–1	Gotland	+		II	11
G20	2014	2	2–1	Gotland		+	II	11
G21	2014	2	2–1	Gotland		+	II	11
G22	2014	2	2–1	Gotland		+	II	11
G11	2014	3	3–1	Gotland		+	I	15
G12	2014	3	3–1	Gotland	+		I	19
U1	2014	4	4–1	Uppland	+		I	18
U2	2014	4	4–1	Uppland	+		I	18
U3	2014	4	4–2	Uppland	+		I	15
U4	2014	4	4–2	Uppland	+		I	15
U5	2014	4	4–3	Uppland	+		I	15
U6	2014	4	4–3	Uppland	+		I	15
U7	2014	4	4–4	Uppland	+		I	19
U8	2014	4	4–4	Uppland	+		I	18
U9	2014	4	4–4	Uppland	+		I	15
U10	2014	4	4–5	Uppland	+		I	15
U11	2014	4	4–5	Uppland	+		I	15
U12	2014	4	4–6	Uppland	+		I	15
U13	2014	4	4–7	Uppland	+		I	15
U14	2014	4	4–8	Uppland	+		I	18
U15	2014	4	4–9	Uppland	+		I	15
U16	2014	4	4–10	Uppland	+		I	15
U17	2014	4	4–11	Uppland	+		I	15
U18	2014	4	4–11	Uppland	+		I	15
U19	2014	4	4–12	Uppland	+		I	18
U20	2014	4	4–12	Uppland	+		I	18
U21	2014	4	4–13	Uppland	+		I	15
U22	2014	4	4–13	Uppland	+		I	15
U23	2014	4	4–13	Uppland	+		I	15
U24	2014	4	4–13	Uppland	+		I	18
U25	2014	4	4–13	Uppland	+		I	18
U26	2012	5	5–1	Uppland	+		I	15
U27	2012	5	5–2	Uppland	+		I	15
U28	2012	5	5–3	Uppland	+		I	15

*P*. *larvae* from colonies owned by three beekeepers (1–3) from the AFB outbreak on Gotland and two beekeepers (4–5) on mainland Sweden were characterized using established ERIC typing methods. The *P*. *larvae* isolates from the apiaries of beekeeper 1, the owner of the first infected apiary, represented both ERIC I and ERIC II genotypes, whereas bacterial isolates from beekeeper 2 and 3 each represented one single genotype, ERIC II and ERIC I, respectively. The *P*. *larvae* isolates from beekeepers 4 and 5, from earlier, unrelated AFB disease incidences in Uppland on mainland Sweden, were all of ERIC I-type (Table 1).

### Core genome MLST

The *P*. *larvae* genomes for both ERIC I and II contain a large number of mobile elements [25] which explains the high number of contigs created in our assemblies (118–291) and the low average N50 value of 60 kb.

The genes of a reference gene target set that were common to all queried genomes (*i*.*e*. those that had a sequence identity≥90% and 100% overlap between query and reference) were established as the shared genome for the cgMLST analysis. When more and more isolates were added to a cgMLST-analysis, the number of core genes used for making the phylogenetic conclusions was often reduced. This reduction was obviously even larger when mixing isolates of both ERIC-types since the shared core genome of these diverse genotypes was smaller than each individual genome. Another reason is that adding more genomes in-and-of-itself, irrespective of ERIC type, can reduce the number of targets that can be included in the analyses, due to random differences and errors derived from both the DNA sequencing and the assembly processes.

**Fig 1:**
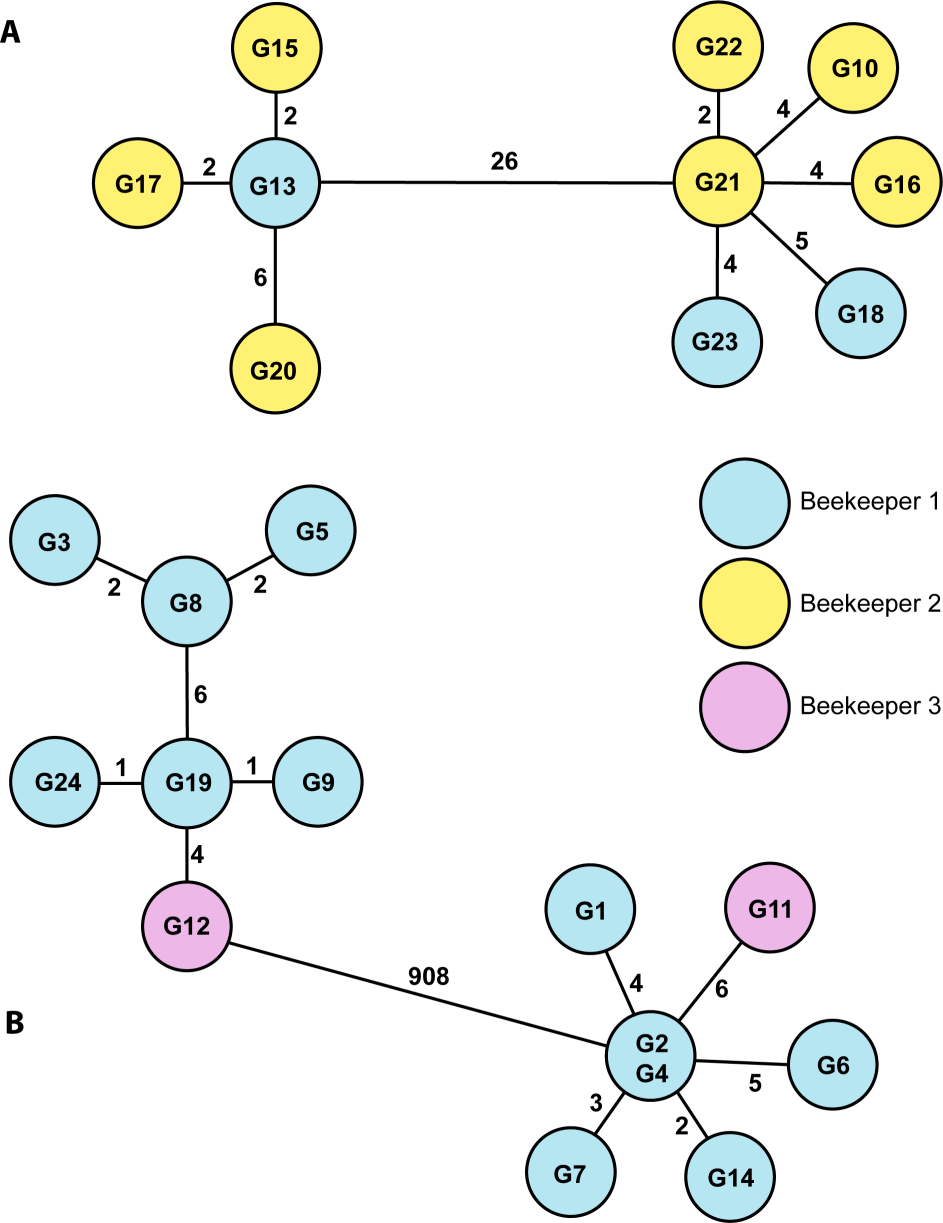
Core genome MLST results of *Paenibacillus larvae* isolates from the outbreak on Gotland in 2014. Two minimum spanning trees generated in SeqSphere+ based on two cgMLST-analysis with ERIC II-isolates (A) and ERIC I-isolates (B), respectively. Results in A are based on 3,309 target genes and B is based on 3,155 target genes. The numbers represent the number of allele differences between isolates. The branch lengths are not proportional to the number of differences. The colors indicate the beekeeper origin of the isolates.

The ERIC I isolates from the Gotland outbreak were analyzed with a gene target set for ERIC I and the ERIC II isolates were analyzed with a gene target set for ERIC II (Fig 1). These analyses show that the ERIC II-isolates formed two groups separated by 26 allele differences between the two groups. Sixty-four (1.9%) of the 3,373 reference targets could not be extracted from the shared genome of the 10 genomes. The ERIC I-isolates clustered in two groups separated with 908 allele differences, meaning that 28.8% of the shared genes were different between the two ERIC I-clusters. In this case 212 out of 3,367 genes (6.3%) could not be used.

There was a low number of core genome allelic differences within the clusters of isolates. Such low amounts of allelic differences strongly suggest a close relation between the isolates and they most likely represent four actual outbreak clones. When including the isolate origin, isolates from beekeeper 1 showed 2–6 allelic differences in each cluster to the nearest isolates from beekeeper 2 and 3 (Fig 1). This is a strong indication that beekeeper 1 was the source of the infection. Considering that beekeeper 1 had provided bees for beekeeper 2 and 3, the most likely scenario is that the *P*. *larvae* isolates from colonies belonging to beekeeper 2 and 3 originated from beekeeper 1.

**Fig 2:**
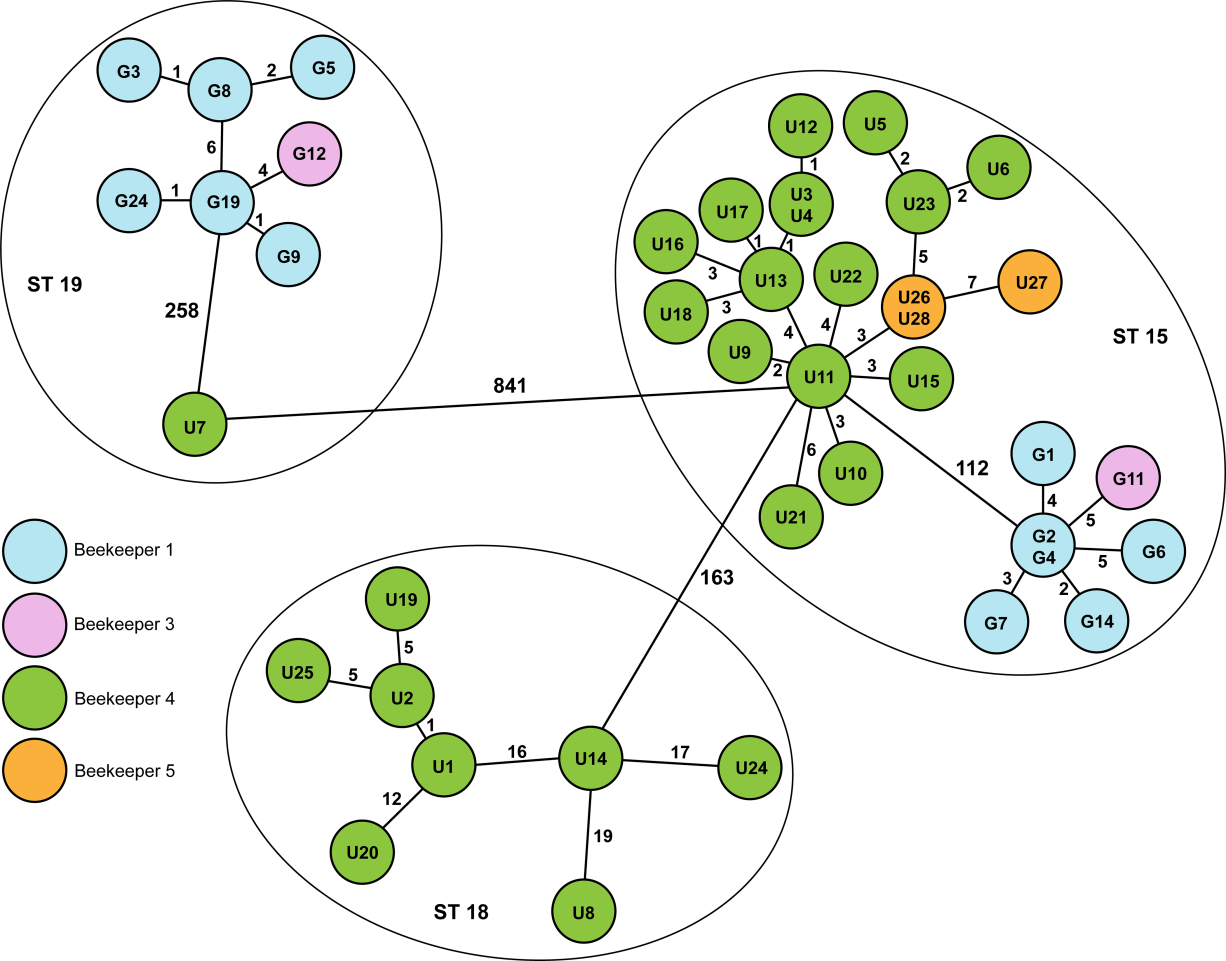
Core genome MLST results of ERIC I-type isolates from AFB outbreaks on Gotland (G) and from Uppland (U). Minimum spanning tree generated in SeqSphere+ based on comparing the 3,052 genes present in the assemblies of the isolates. The numbers represent the numbers of allele differences between groups of isolates. The lengths of the lines are not proportional to the number of differences. The colors indicate the origin of the isolates (the beekeeper/owner of the apiaries). The circles show which MLST sequence type the isolates belonged to. The minimum spanning tree should not be over-interpreted as showing transmission routes, only the level of relatedness.

To further analyze the diversity within the MLST-types and ERIC-types and between isolates from the same apiary, the 14 ERIC I isolates from Gotland were analyzed together with the 28 ERIC I isolates from mainland Sweden. *Paenibacillus larvae* isolates of ERIC II genotype were isolated from the Gotland samples only (Table 1). The number of shared genes in the ERIC I core genome was 3,052, *i*.*e*. 9.4% of the genes were disregarded. The resulting minimum spanning tree (Fig 2) shows that the ERIC I-isolates formed five groups of which one consisted only of isolate U7. However, the U7 isolate had the same MLST type (ST 19) as one of the clusters from the Gotland outbreak even though there were 240 allele differences between them. That means that 8.4% of the genes included in the analyses had one or more mutations among the different isolates while still sharing the same MLST type. The ST-15 isolates also formed two distinct clusters separated by 112 allele differences.

When analyzing ERIC I and ERIC II isolates in the same analysis, the WGS-results split ERIC I and II isolates into two clear and consistent clades separated by 1,493 allele differences, or by 52% of the shared genes when using the gene target set derived from the ERIC II genome (data not shown). The ERIC I isolates were also internally very genetically diverse which is consistent with previous studies [8].

Most of the U-isolates created one big cluster of high genetic similarity (ST15) and this clone was the dominant clone in the apiaries of beekeeper 4. Since these apiaries were separated by tens of kilometres, far beyond the normal flying range of bees, the implication is that the contamination spread between these apiaries via the transfer of beekeeping material. The same clone was also the only clone found in samples from beekeeper 5 who for many years had acquired bees and material from beekeeper 4 who, in turn, has a long history of AFB.

The ST 15-cluster gives some insight into the natural diversity of a clone that has spread to apiaries managed by the same beekeeper. The number of differences between isolates inside that cluster was between 0–16. Isolates U8 and U7 represent an example of two distinct genotypes found in the same apiary. The two isolates U26 and U28 from beekeeper 5 were isolated in 2012 and showed only three allele differences to isolate U11 from beekeeper 4. The fact that U26 and U28 were isolated in 2012 and U11 two years later indicates that the genomes are relatively stable over time. The isolates U1, U8, U14, U20 and U24, all isolated in 2014, were separated by 12–19 allele differences and they represent an example of greater diversity of a clone between different apiaries.

## Discussion

This study presents the use of WGS and core genome MLST for separating *P*. *larvae* isolates with high resolution and the application of these techniques to trace the progress of two separate American foulbrood epidemics in Sweden. The study clearly separates the *P*. *larvae* isolates from the 2014 outbreak of AFB on the island Gotland into four different clones. The high degree of similarity between the clones strongly support that they were directly related through transmission.

When using a cut-off value of CT (Cluster Threshold), ≤ 10 the Gotland ERIC II isolates divide into two separate clusters but when setting a cut-off value of CT ≤ 30, the same isolates represent one clone or cluster. Since we demonstrated that the natural diversity of isolates within a large beekeeping practice was 16 allele differences at the most, it is likely that the more accurate cut-off value for what represents an outbreak clone or cluster is closer to 10 than 30. However, there is no consensus on how similar or closely related two bacterial isolates must be to establish that they originate from the same outbreak. The number of allele differences may vary with the lifecycle of the bacterium, the time-frame of sampling (longer infection time possibly leads to more allele variants), the mutation frequency of the bacterium and even which individual bacterial colony was chosen and picked from the agar plate. The high number of variables makes setting cut-off values difficult but studies on other bacteria have attempted to do this by investigating confirmed outbreak isolates. A study using cgMLST on confirmed outbreaks of *Listeria monocytogenes* set the threshold for the isolates to be part of the same cluster, the so-called to ≤ 10 allelic differences [26]. Another study of methicillin-resistant *Staphylococcus aureus* (MRSA) outbreaks selected a cutoff of ≤ 30 SNP differences to indicate a putative transmission event [27].

Another factor affecting cut-off values is the number of loci used for analysis. A cgMLST-analysis of 1,000 loci could therefore have 1/3 of the cut off value for an analysis of 3,000 loci. In the current analysis however, most of the target genes were part of the shared genomes.

The low allelic differences in this study between outbreak isolates within the same cluster clearly show their strong relatedness. Such low diversity estimates among outbreak isolates have been observed during disease outbreaks of other bacterial species [27,28] and also when multiple bacterial isolates from one individual animal that died from anthrax, a disease caused by another spore-forming bacterium *Bacillus anthracis* [14], were compared. By these criteria, approximately 10 allelic differences between isolates should be enough to define a cluster of a single bacterial clone.

The results presented here are based on a genome-wide, gene-by-gene approach, similar in concept to the traditional 7-gene MLST but extended to the whole genome. A full SNP analysis would increase the resolution even more since SNPs are analyzed individually rather than used to define the allelic status of whole genes and SNPs that lie outside the coding regions can be included. However, it is not clear that such additional resolution would significantly improve the accuracy of the tracing or contribute more noise to the data and analyses. One advantage of cgMLST over SNP-based analyses is that new isolates can be added progressively without requiring a re-calibration of the database, making it easier to compare and mix different studies. SNP-based analyses can always be performed on the same genomic data if additional resolution is required.

When creating a cgMLST target set, it is advantageous to have access to a complete genome. The only complete, fully annotated *P*. *larvae* genome available at the time this study started was DSM 25430 (NCBI accession NC_023134), which is an ERIC II-genome. However, the ERIC I genome (DSM 25719, NCBI WGS accession ADFW) is considerably larger than the ERIC II genome (4.5 Mb vs 4.0 Mb) [25]. This means that additional allelic differences are potentially available among the ERIC I genomes when the target set is defined from an ERIC II-genome. One possible way to remedy this would be to create an ERIC I-gene target set from the large amount of sequence data produced. However, later the ERIC I-genome of ATCC 9545 (NZ_CP019687) was published and the full ERIC I genome could be used by a gene target set created from this genome. The difference between analyzing our ERIC I-genomes using the ERIC I reference instead of the ERIC II reference was however minor (data not shown) and did not change any of the conclusions drawn from this study.

The cgMLST method is based on a ‘shared genome’ concept [29] where the allelic differences between isolates are distilled into a reduced set of alleles that all isolates have in common. The disadvantage is that with increasing divergence among all isolates fewer common alleles are identified for priming the analyses. The size of the shared genome is also affected by technical factors such as sequencing coverage, assembly quality and genetic deletions. For example, the original 3,373 gene targets, available from the ERIC II reference genome, was reduced to 3,309 for the analyses of the ERIC II isolates in this study. This level of resolution was more than sufficient to cluster the isolates accurately by both their ERIC and ST classifications with further significant clusters identified with each of these and was a significant improvement on ERIC typing or 7-gene MLST.

The results presented in Fig 1 suggest, but do not conclusively determine, that beekeeper 1 is the source of the outbreak. Instead of being the source, beekeeper 1 could theoretically have received three to four different clones of *P*. *larvae* from beekeepers 2 and 3. Fig 1 indicates that the isolates were strongly connected but the direction of the transmissions needed confirmation (the same as for other typing techniques) by more epidemiological links and background information. However, in combination with the information that there had been a transfer of bees from beekeeper 1 to beekeepers 2 and 3, the genomic results in this study supported the conclusions on the origin of the outbreak. The bacterial isolates from earlier AFB outbreaks on mainland Sweden unrelated to the Gotland outbreak served as a reference to determine the diversity of clones and spread within beekeeping operations managing several apiaries.

As increasing numbers of whole genomes of *P*. *larvae* become available, a standardized public cgMLST database capturing the diversity of *P*. *larvae* will facilitate future analyses of AFB epidemics.

## Conclusions

We have used WGS to look at the spread of American foulbrood during outbreaks in Sweden. This is, to our knowledge, the first time WGS of *P*. *larvae* has been used for an epidemiological investigation. We found that this method showed higher discriminatory power than previously described methods used for AFB epidemiological studies and it had the necessary resolution to draw conclusions on the origin of a disease outbreak and shows great promise to be a future standard for epidemiological studies on this devastating disease.

## Supporting information

S1 Table Core genome-MLST targets for ERIC I strains. The targets were extracted from *P*. *larvae* ATCC 9545 (ERIC I strain).
(XLS)

S2 Table Core genome-MLST targets for ERIC II strains. The targets were extracted from *P*. *larvae* DSM 25430 (ERIC II strain).
(XLS)
